# US President Warms Up as Genome Tempers Cool

**DOI:** 10.1100/tsw.2001.10

**Published:** 2001-03-09

## Abstract

Nature leads this week with a story about hints that the Bush administration may be softening its position on global warming. Science kicks off with a story about how an effort to sequence the rat genome makes for strange bedfellows.

Christine Todd Whitman, the newly appointed head of the Environmental Protection Agency, seems to be learning from her mistakes. Soon after her nomination was announced earlier this year, she was roundly criticized for misstatements about the causes of global warming. Whitman must have done her homework. This week in an interview on the television network CNN, she said, "The science is good on global warming," reports *Nature* in its lead story. Although she stopped short of endorsing the 1997 Kyoto Protocol - an unratified agreement by several industrialized countries to reduce greenhouse emissions by five percent before 2012 - Whitman claimed to support limits on carbon emissions, particularly from power plants, says *Nature*. The interview was the clearest sign yet that the Bush administration is adopting a surprisingly friendly attitude towards the environment. Other cabinet members, including Secretary of State Colin Powell and Treasury Secretary Paul O'Neill have recently spoken about the need to address climate change, reports *Nature*. Although she doubts that Bush has a coherent strategy in place, *Nature* reports that Eileen Claussen, president of the non-profit Pew Center on Global Climate Change, welcomed the administration's renewed emphasis on global warming. President Clinton lost interest in the subject during the last years of his administration, says *Nature*. Which just goes to show - there is more than one way to heat up the White House. *Science* did not cover this story.

Less than a month after their sometimes rancorous race to sequence the human genome ended in a tie, the publicly funded National Human Genome Research Institute (NHGRI) and the private company Celera Genomics are at it again, reports *Science* in their lead story. But this time, they are joining forces to sequence the Norway brown rat. The NHGRI and the National Heart Lung and Blood Institute will provide the money, the genome center of the Baylor College of Medicine will lead the sequencing project, and Celera will contribute brute-force sequencing and "advice", reports *Science*. Several smaller groups will also participate. The unexpected alliance has set an ambitious goal for itself: a gap-free rat genome sequence by 2005 for less than $100 million and with half the redundancy of the human genome sequence. Celera founder Craig Venter is doubtful that they can sequence the whole rat genome at that level of redundancy, says *Science*, but the leader of the Baylor group, Richard Gibbs, thinks they can do it. Regardless, researchers are "delighted" by the project, reports *Science*. And they won't have to pay to see the results. In another first for Celera, the entire rat genome will be released publicly. *Nature* did not cover this story.

Both *Science* and *Nature* reported that space scientists are claiming that a chain of crystals found in a meteorite is evidence for life on Mars. The scientists say the chain is too regular to be produced by non-biological processes. Most scientists remain skeptical, to say the least.
